# Two case reports of unexpected tracheal agenesis in the neonate: 3 C’s beyond algorithms for difficult airway management

**DOI:** 10.1186/s12887-017-0806-z

**Published:** 2017-02-08

**Authors:** Beate Grass, Leopold Simma, Michael Reinehr, Urs Zimmermann, Claudine Gysin, Georg Henze, Vincenzo Cannizzaro

**Affiliations:** 1 Department of Intensive Care Medicine and Neonatology, University Children’s Hospital Zurich, Steinwiesstrasse 75, 8032 Zurich, Switzerland; 2 Department of Pathology, University Hospital Zurich, Raemistrasse 100, 8091 Zurich, Switzerland; 3 Department of Neonatology, Hospital Buelach, Spitalstrasse 24, 8180 Buelach, Switzerland; 4 Department of Otorhinolaryngology, University Children’s Hospital Zurich, Steinwiesstrasse 75, 8032 Zurich, Switzerland; 5 Department of Anesthesia, University Children’s Hospital Zurich, Steinwiesstrasse 75, 8032 Zurich, Switzerland; 6 Children’s Research Center, University Children’s Hospital Zurich, Steinwiesstrasse 75, 8032 Zurich, Switzerland

**Keywords:** Case report, Tracheal agenesis, Unexpected difficult airway, Communication, Culture, Capnography

## Abstract

**Background:**

Handling neonates with postnatal respiratory failure due to congenital airway malformations implies knowledge about emergency management of unexpected difficult airway. In these stressful situations both technical and communication skills of the caretakers are essential.

**Case presentation:**

Two cases with prenatally unknown tracheal agenesis are reported.

**Conclusion:**

In the presented cases, airway malformation and subsequent difficulties upon endotracheal intubation were not adequately communicated between caretakers. We discuss the aspects of culture, communication, and capnography.

## Background

Tracheal agenesis is one of the rarest congenital airway malformations. It was first described by Payne ([1] Payne) in 1900 and later classified in three types by Floyd ([2] Floyd). An incidence of 1:50 000 to 1:100 000 births with male preponderance is reported in the literature ([3] Klotz, [4] van Veenendal). In most cases other malformations are associated ([4] van Veenendal, Evans [5]). Affected neonates present with severe refractory respiratory distress after delivery. Even in neonates with prenatal diagnosis of tracheal agenesis, postnatal airway management is challenging. Despite intensive treatment overall prognosis of this malformation is poor and surgical curative attempts are still experimental ([4] van Veenendal, [6] Hartnick). We report two cases with antenatally unknown tracheal agenesis. Importantly, both neonates were supposedly endotracheally intubated prior to take-over. Correct endotracheal intubation was also supported by positive capnography. Personal and professional skills training might be a way to counteract the triad of negative safety culture, bad communication, and false positive capnography results.

## Case presentation 1

The 24-year-old gravida 1 para 1 mother had her first pregnancy check at 25 weeks of gestational age (GA) and polyhydramnios was detected. At 30 weeks and five days GA a prolapse of the amniotic sac occurred and antenatal steroids were administered to induce lung maturation. During the fetal assessment a major cardiac malformation was diagnosed. At 31 weeks GA a male infant was delivered by caesarean section due to active labour and breech presentation. The infant was severely depressed at birth, with a 1-min Apgar score of 3. He required bag-mask ventilation and was intubated orally with significant difficulty due to impaired vision at 4 min with a 2.5 mm endotracheal tube (ETT). At 5 and 10 min the Apgar was scored 4 and 5, respectively. Following administration of surfactant the oxygenation improved significantly, the neonate was cardiopulmonary stable on low respirator settings and the outborn patient was transferred to our neonatal intensive care unit. The birth weight was 1230 g (10^th^–25^th^ percentile). Physical examination was remarkable for anal atresia, single transverse palmar crease on the right hand and bilateral clinodactyly. The diagnostic work-up included echocardiography, X-ray films, and an abdominal ultrasound showing a balanced double outlet right ventricle, a double-bubble sign and dysplastic S2/S3 vertebrae, and an absent right kidney, respectively.

**Fig. 1:**
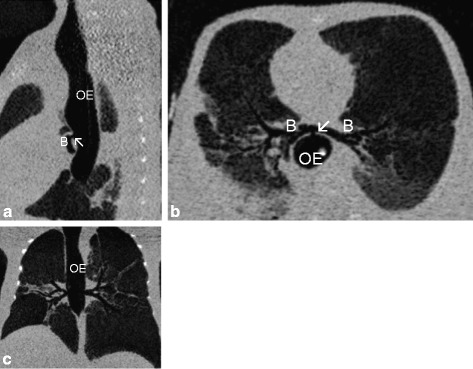
Chest CT scans (sagittal (**a**), horizontal (**b**) and axial (**c**)) show the absence of the trachea and bronchi (B) arising from the oesophagus (OE) via a fistula (arrow ↖)

On the second day of life, the oral ETT was replaced by a nasal ETT due to a significant leak and worsening gas exchange. This elective nasal intubation turned out to be challenging since the vocal cords could not be clearly visualized. However, end-tidal CO2 was positive, chest excursions and breath sounds were present, and oxygen saturation was normal. The difficulty upon intubation was not communicated in the team. Due to a persisting significant air leak of the 2.5 mm ETT on day of life 6 another exchange to a 3.0 mm ETT was planned. Upon inspection of the larynx vocal cords could be visualised, however the cords were fused and no tube could be passed while ventilation via bag-mask was possible. Hence, we called for an urgent endoscopy of the airway which showed a fistula in the distal oesophagus, opening with every inflation delivered. Computed tomography (CT) of the chest showed complete absence of the trachea and bronchi arising from the oesophagus, known as Floyd Type II tracheal agenesis (Fig. 1). Due to the multiple malformations (airway, cardiac, intestinal) and extremely unfavourable prognosis combined with prematurity, intensive therapy was withdrawn with parental consent. Unfortunately, consent to an autopsy could not be obtained.

## Case presentation 2

**Fig. 2:**
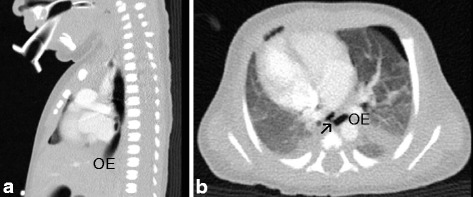
**a** Sagittal chest CT scan showing long-segment agenesis of the trachea, only oesophagus (OE) visible. **b** Horizontal chest CT scan displaying the fistula (arrow ↗) from the oesophagus (OE) to a ventral distal tracheal pouch (blind proximal ending) at the level of thoracic vertebrae 4–5

An outborn male neonate was delivered vaginally at 39 weeks and 2 days GA to a 34-year old gravida 2 para 2 mother. A neonatologist attended birth due to intrauterine diagnosed polyhydramnios and dextrocardia with normal four-chamber-view in fetal echocardiography. The infant developed severe dyspnea immediately after birth requiring intubation. Correct position of the endotracheal tube was confirmed by positive capnography. Apgar scores were 1/3/5 at 1, 5, 10 min, respectively. The birth weight was 3500 g. Our neonatal transport team was informed due to persistent ventilation problems and varying oxygenation resulting in severe combined acidosis. Suspicion of cyanotic congenital heart defect was raised and prostaglandine infusion was started. Upon arrival, we encountered a severely depressed neonate with oxygen saturation of 30% on mechanical ventilation with high inspiratory pressures and fraction of inspired oxygen (FiO2) of 1.0. To verify correct endotracheal intubation laryngoscopy was performed revealing oesophageal intubation, while the larynx appeared atretic. Indeed, endotracheal intubation could not be achieved. Eventually, hypoxemia led to bradycardia requiring two courses of CPR and high doses of catecholamine support. After placement of a laryngeal mask adequate oxygenation was achieved while ventilation only improved slightly and severe acidosis persisted. Anal atresia without fistula and cryptorchidism was diagnosed clinically. Upon arrival in our tertiary neonatal center, endoscopy revealed laryngeal atresia with an oesophageal fistula to the respiratory system. The CT scan (Fig. 2) confirmed laryngeal atresia and showed long-segment agenesis of the trachea with a fistula from the oesophagus to a distal tracheal pouch (blind proximal ending) at the level of thoracic vertebrae 4–5 and tracheal bifurcation at thoracic vertebra 6, known as Type I tracheal agenesis according to the Floyd classification. Echocardiography confirmed dextrocardia with otherwise normal cardiac structures. Chest and abdominal X-ray showed fusion vertebrae, only 11 pairs of ribs, bony changes of the sacrum and pelvis. Cerebral ultrasound showed cerebral edema. The neonate met the criteria of hypoxic ischemic encephalopathy regarding the resuscitation details and the neurological assessment with a Thompson score of 10. Due to both the absence of a non-experimental therapeutic option to establish a functional airway and severe hypoxic ischemic encephalopathy we redirected care.

**Fig. 3:**
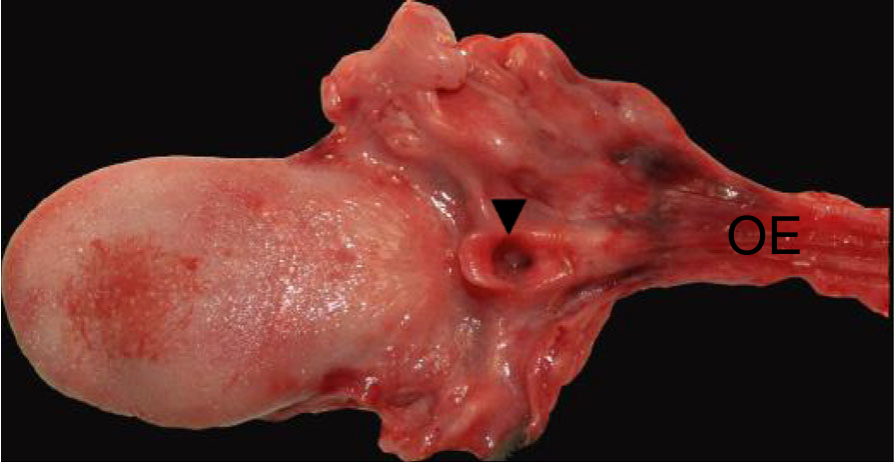
Surface of the tongue with view directly into the open, blind ending larynx (▼). Tissue at the right side just contains parts of the oesophagus (OE), no trachea

**Fig. 4:**
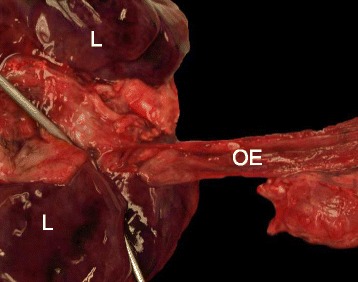
Oesophagus (OE) coming from the right side to the lung’s backside (L). The two metal testing probes are inserted into the two main bronchi (entering through the oesophageal fistula)

**Fig. 5:**
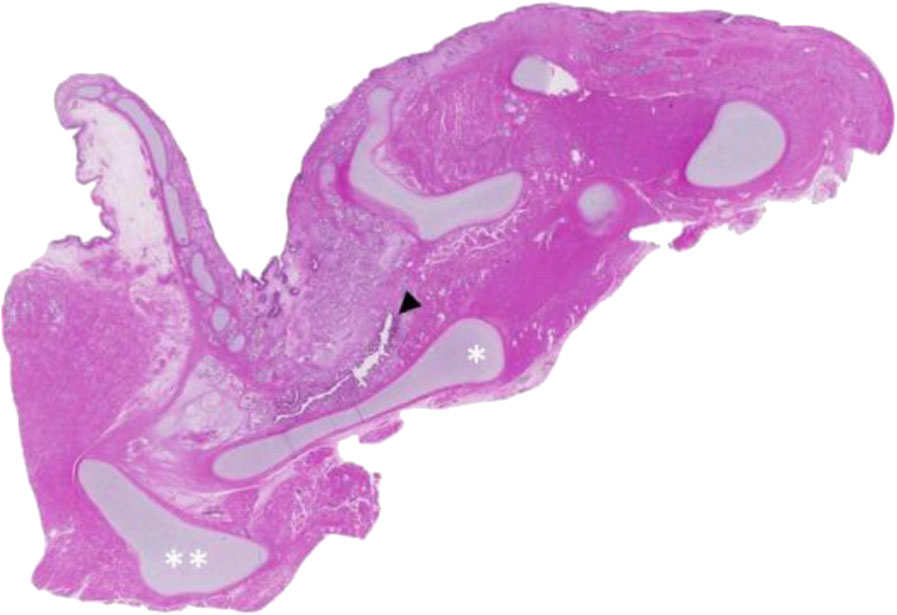
Histological sagittal cut through the larynx (H&E, 1.25x) with the opened epiglottis (* thyroid cartilage, ** hyoid). The arrow head (◀) points onto the blind-ending larynx (trachea should normally join to the right)

**Fig. 6:**
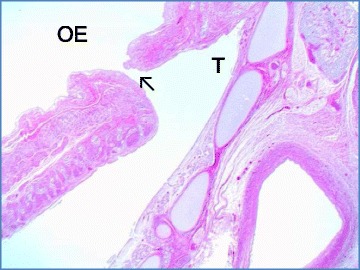
Left upper edge: Oesophagus lumen (OE). The small fistula (arrow ↖) to the blind ending tracheal (T) pouch (middle-right upper side) can be seen. Normal structured main bronchi with hyaline cartilage inside the wall (H&E, 2.5x)

In this case, autopsy was performed and confirmed tracheal agenesis. Laryngeal atresia (Fig. 3) was found with total loss of cartilage and connective tissue for the whole segment between larynx and tracheal bifurcation. Both principal bronchi emerged from the distal trachea with a proximal closed pouch (Floyd Type I; Fig. 4). The tracheal pouch itself showed a fistula to the esophagus which was capable of delivering small amounts of oxygen to the lungs during ventilation through a laryngeal mask. Histologically, a blind ending of the larynx at the thyroid level was verified (Fig. 5). A cut section through the fistula showed a proximally blind-ending laryngeal pouch with overlying respiratory mucosa passing over into a normally structured system of main bronchi (Fig. 6). We confirmed anal atresia and additionally found an incomplete segmentation of the right lung. Because of the severe hypoxia acute neuronal and myocardial necrosis as well as petechial bleeding in serous membranes showed at the autopsy. In the end, cardiorespiratory failure occurred due to acute severe hypoxic injury of heart and brain.

## Discussion and conclusions

Our focus in these two reports is on the importance of “3 C’s” beyond algorithms for difficult airway management, namely, the aspects of culture, communication, and capnography. The presented cases emphasize the importance of classification and communication of a difficult airway in neonates with postnatal severe respiratory distress.

Laryngeal atresia and tracheal agenesis are rare congenital conditions that result from the failure of the larynx and trachea to re-canalize during embryogenesis ([6] Hartnick, [7] Ambrosio, [8] Ahmad). Some of the traits are stronger male preponderance, significantly higher rates of premature birth ([4] van Venendaal), more complex heart defects, and intestinal and renal abnormalities. Malformations with tracheal agenesis have common genetic characteristics, but are different from tracheo-oesophageal fistula malformations found with VACTERL-association. Tracheal agenesis can be one malformation in a so-called TACRD (tracheal atresia, complex congenital cardiac abnormalities, radial ray defects, duodenal atresia) association ([5] Evans).

Prenatal diagnosis via ultrasound remains difficult in the presence of an oesophageal-bronchial fistula. In the absence of a fistula, Congenital High Airway Obstruction Syndrome (CHAOS) can be diagnosed in some cases ([9] Groot-van der Morren). Under optimal conditions prenatal ultrasound detects polyhydramnios ([4] van Veenendal), fetal hydrops, hyperechogenic enlarged lungs, a flattened or inverted diaphragm, and a fluid-filled dilated airway distal to the obstruction ([8] Ahmad, [9] Groot-van der Morren, [10] Sanford, [11] Oenderoglu). In context of unexplained polyhydramnios associated with congenital malformations suspicion of tracheal agenesis should be raised. In these cases, exploration of the airway by fetal MRI should be considered ([12] Bertholdt). To plan a safe delivery, the ex utero intrapartum treatment (EXIT-procedure) has been shown to be a useful management strategy for the anticipated difficult airway ([6] Hartnick, [10] Sanford, [13] Vaikunth). During the EXIT-procedure, either a (temporary) surgical airway is established or ECMO cannulation is performed to bridge to a later surgical repair.

Respiratory distress and strong respiratory effort in an aphonic cyanotic neonate is the most frequent presentation of tracheal agenesis ([14] Dijkman). In presence of an oesophageal-bronchial fistula the infant can be stabilized by bag mask-ventilation and oesophageal intubation or insertion of a laryngeal mask ([15] Vanzati). In some cases of tracheal agenesis, emergency tracheotomy has been performed, but the procedure is seldom and complex ([16] Krause, [17] De Luca). Overall, tracheal agenesis has a very poor prognosis, even though cases of surviving patients with experimental therapies have been reported in the literature ([18] Hiyama, [19] Soh, [20] Tazuke).

After making the diagnosis of tracheal agenesis, we discussed the cases with all the physicians who were involved in the management. The attending neonatologist who performed the intubation in the delivery room in the first case reported that visualisation of the larynx was difficult due to secretions. Following intubation, thorax excursions and improvement of gas exchange were observed. Moreover, subsequent administration of surfactant resulted in an additional stabilization of the neonate. Hence, the initial doubts regarding correct ETT tube positioning vanished. Nonetheless, “difficult tracheal intubation” was mentioned at hand-over. The next 24 h were characterized by the observation of both a significant airway leak despite adequate ETT size and tracheal suctioning never yielding normal tracheal aspirates but rather saliva-like mucous. Without mentioning particular findings, a paediatric intensivist electively replaced the oral ETT by a nasal ETT. Again, positive capnography was taken as correct tube positioning despite medical history and lacking vision of the typical landmarks during intubation. Both airway classification and difficult airway were not communicated in the team. In the second case, no irregularities with regard to intubation were described. Positive capnography and initial improvement of the neonate were also interpreted as correct endotracheal intubation. We assume that massive air leakage of the oesophageal tube led to worsening oxygenation and insufficient ventilation. Only after placing a laryngeal mask, sufficient air exchange via tracheo-oesophageal fistula was achieved.

In the depicted cases, the visibility of the landmarks upon intubation was clearly impaired and tracheal intubation was not under vision. However, positive capnography reassured the clinicians of correct endotracheal intubation. Positive capnography (end tidal CO2) measurement was achieved by inadvertent oesophageal intubation and ventilation via the oesophageal fistula to the bronchial system. Although end tidal CO2 is intended to prevent mal-positioning of the endotracheal tube, our cases demonstrate that this is not always true for rare congenital anomalies. Positive capnography can thus be misused in two ways. First, in reassuring oneself despite pathologic view and landmarks and second, to justify correct intubation towards others.

Based on the two cases, we discussed how to proceed and manage future patients. In case of antenatal suspicion of airway malformation, intrauterine referral to a tertiary center would be strongly recommended. Diagnostic imaging via fetal MRI has the potential to demonstrate tracheal agenesis and allow for timely interdisciplinary discussion and counselling of the parents. In case of postnatal suspicion at the referral hospital, securing the difficult airway via laryngeal mask or oesophageal intubation (tracheoesophageal fistula) is mandatory and the priority. After that, expeditious transfer to a tertiary center for urgent diagnostic assessment such as medical imaging and endoscopy of the airway should be organised, followed again by an interdisciplinary case discussion.

In summary, use of airway classification should be encouraged in all specialities dealing with endotracheal intubation. It is routine for anaesthesiologists to formally assess the airway and to communicate airway classification at hand-overs. In contrast, this is not always the case among paediatric intensivists and neonatologists. One likely explanation for this is that broad experience leads to the confidence in naming abnormalities encountered during routine procedures. Describing irregularities upon intubation can thus be seen as an indication of a fund of experience. Ignoring facts inconsistent with a favoured hypothesis, overemphasising positive findings, and discounting negative findings is a known problem in the psychology of clinical problem solving ([21] Elstein). An environment in which team members can speak up using critical language to express concerns promotes good communication culture ([22] Leonard). These human factors in addition to correct use and interpretation of technical devices such as capnography help maximizing patient safety.

## Abbreviations

CHAOS: Congenital High Airway Obstruction Syndrome
ECMO: Extracorporeal membrane oxygenation
ETT: Endotracheal tube
EXIT: Ex utero intrapartum treatment
GA: Gestational age
TACRD: Tracheal atresia, complex congenital cardiac abnormalities, radial ray defects, duodenal atresia
